# Nipple-Sparing Skin-Reducing Mastectomy and Immediate Prepectoral Breast Reconstruction: A New Surgical Approach

**DOI:** 10.1093/asjof/ojaf133

**Published:** 2025-11-04

**Authors:** Diletta Maria Pierazzi, Ulpjana Gjondedaj, Marco De Prizio, Alessandro Neri

## Abstract

**Background:**

Nipple-sparing mastectomy in medium-large, ptotic breasts presents challenges because of redundant skin and risks to the nipple–areola complex (NAC). Advances in skin-reducing techniques and immediate prepectoral reconstruction aim to improve both oncological safety and aesthetic outcomes.

**Objectives:**

The authors of this study present a novel surgical approach designed to enhance NAC vascularization by performing a nipple-sparing skin-reducing mastectomy exclusively through the lower lateral triangle of a Wise-pattern incision with immediate prepectoral polyurethane-coated breast implant (Microthane, POLYTECH, Dieburg, Germany) reconstruction. This approach seeks to minimize implant exposure risks by limiting the scar laterally, thus optimizing breast contour and patient satisfaction.

**Methods:**

A prospective analysis of 10 patients undergoing monolateral procedures was conducted between April and August 2023. Inclusion criteria included Grade 2 ptosis with an expected nipple repositioning of ≤8 cm. The technique preserved vascular connections through precise de-epithelialization and assessed intraoperative flap perfusion. Implant selection ensured symmetry. Postoperative outcomes were evaluated using the BREAST-Q questionnaire and routine follow-ups (18 months). Associations between BREAST-Q scores and patient characteristics were analyzed through Pearson correlation and analysis of variance.

**Results:**

Mean patient age was 52 years, BMI 24 kg/m^2^, and implant volume 328 cc. Complication rates were low, with no cases of capsular contracture or implant displacement. BREAST-Q scores demonstrated high satisfaction, and a significant positive correlation was found between BMI and physical well-being (*P* = .03).

**Conclusions:**

This single-stage technique for immediate prepectoral breast reconstruction in ptotic breasts may be safe and provide good aesthetic and functional outcomes. Further studies with extended follow-up and greater numbers are warranted.

**Level of Evidence: 4 (Therapeutic):**

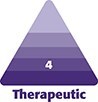

Implant-based breast reconstruction after nipple-sparing mastectomy (NSM) is nowadays considered an oncologically safe and reliable option in many breast cancer patients; it is associated with good cosmetic results and better quality of life.^1^ However, large and ptotic breasts present significant difficulties in surgical management, as the skin envelope may be redundant, and the removal of the supportive breast tissue underneath may damage the nipple–areola complex (NAC). Thus, these patients with notably large and ptotic breast often experience complications such as nipple necrosis or dislocation and persistent edema of the skin flaps, with unsatisfactory outcomes when NSM is performed. Nava et al described in 2006 a new technique called “skin-reducing mastectomy” that consists of a Wise-pattern mammoplasty incision with a de-epithelialized dermal sling and direct submuscular implant insertion.^2^ This procedure allows reconstruction of medium-large and ptotic breasts without the need for myocutaneous pedicled or free flaps. The Wise-pattern incision has been proposed in patients with medium-large and ptotic breasts because it allows the preservation of the skin around the NAC, lifts and cones the breast into a less ptotic shape, and removes skin redundancy.^3^ Moreover, prepectoral reconstruction with or without acellular dermal matrix (ADM) is currently considered a common and safe technique, allowing to overcome several complications of subpectoral implants, such as capsular deformity, implant displacement, reduction in strength of pectoral muscles, loss of muscle function, and chronic pain.^4^ In addition, the current trend is to perform breast reconstruction simultaneously with oncological surgery rather than a 2-stage reconstruction with tissue expanders, avoiding 2 operative steps and several follow-up visits.^5-8^

In this study, we describe an original technique of nipple-sparing skin-reducing mastectomy through the lower lateral triangle of a Wise pattern with an immediate prepectoral reconstruction polyurethane-coated anatomical implant (Microthane, POLYTECH, Dieburg, Germany) preserving the vascular dermal connections as much as possible. The purpose of the authors of this study is to illustrate our findings with a new surgical procedure of mastectomy combined with simultaneous removal of excess breast skin in individuals presenting medium-large and ptotic breasts, performing an immediate prepectoral implant-based reconstruction, reporting technique reliability, and the satisfaction level of the patients.

## METHODS

We conducted a prospective data collection including all the nipple-sparing skin-reducing mastectomies through the lower lateral triangle and prepectoral breast reconstructions performed between April 2023 and August 2023.

Overall, we performed 10 nipple-sparing skin-reducing mastectomies combined with immediate direct-to-implant prepectoral breast reconstructions using anatomical polyurethane-coated implant (mono-lateral procedure). The anatomical implant was positioned directly beneath the skin, without additional coverage. The patients were administered the Breast-Q score to evaluate breast satisfaction, postoperative results, and physical well-being.^9^ This study was carried out in compliance with the ethical standards in the Declaration of Helsinki. A detailed written informed consent was given to the patients.

### Patient Selection

All patients who met the criteria outlined below were eligible for the proposed procedure. The inclusion criteria were patients with Grade 2 breast ptosis (defined as breasts in which the nipple position is located below the inframammary fold). The degree of ptosis was calculated according to Regnault's classification, with an expected rise in NAC position after NSM skin reducing of no more than 8 cm, in which a prepectoral reconstruction can be performed.^10^ At presurgical evaluation, an adequate breast tissue coverage on the digital mammogram of at least 1 cm, corresponding to a Class 2 or 3 according to Rancati classification, was considered necessary for inclusion in the study.^11^

Exclusion criteria consisted of patients with Stage IV breast cancer or considered to have a high risk of recurrence, skin and/or nipple involvement, and those presenting comorbidities including uncontrolled glycemia, severe obesity, and chronic immunosuppression. Active tobacco use is not considered a contraindication for our procedure.

### Surgical Technique

**Figure 1.**
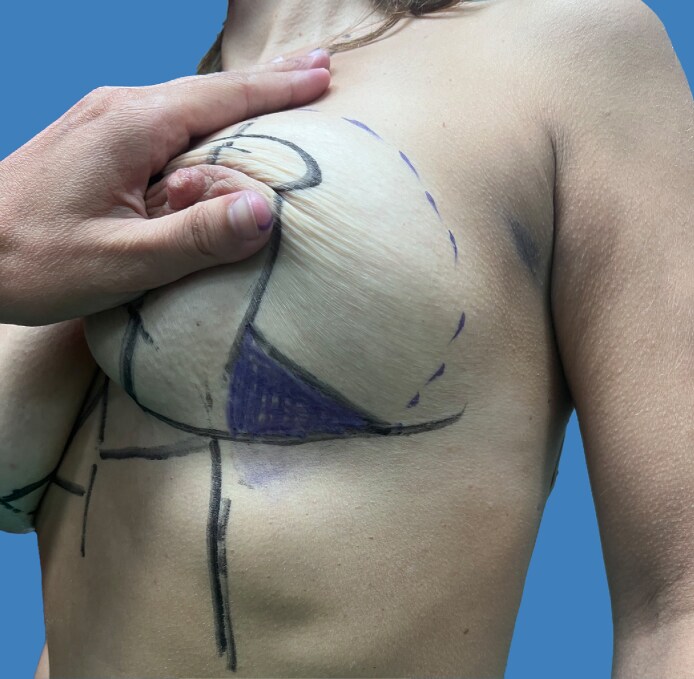
A 44-year-old female patient. The preoperative drawing was carried out according to the rules of Wise-pattern marking: the incision, where the oncology surgeon performed the mastectomy, was marked from the lower lateral triangle of Wise pattern.

**Figure 2.**
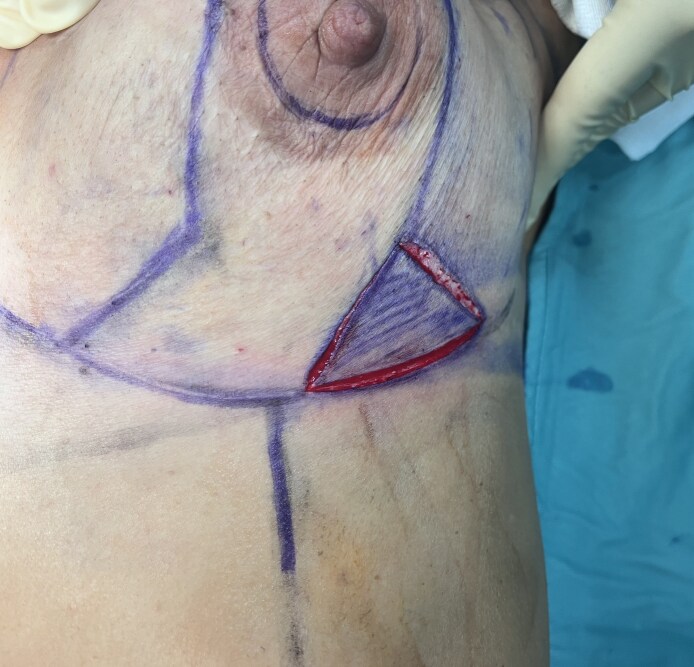
A 44-year-old female patient. The oncology surgeon undertook the mastectomy from the lower lateral triangle of the Wise pattern.

**Figure 3.**
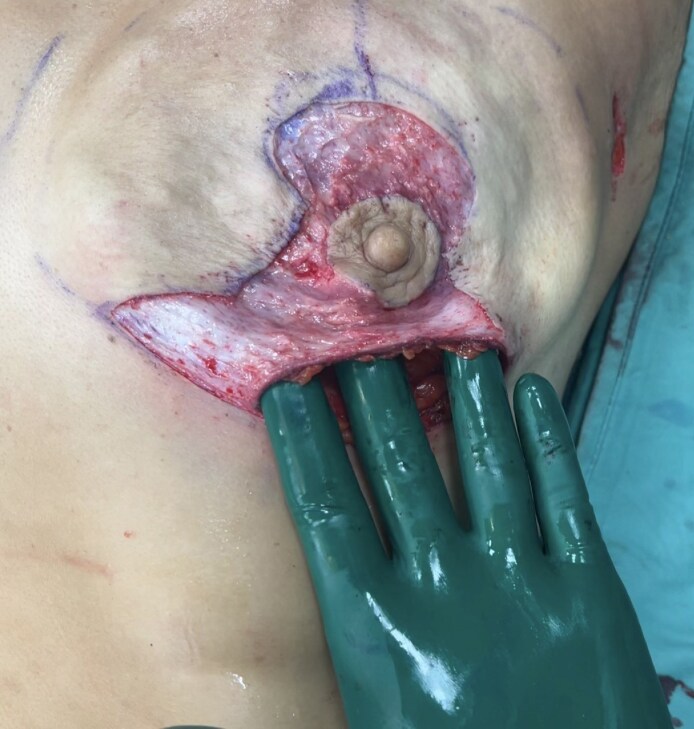
A 44-year-old female patient. The portion of skin remaining within the Wise-pattern design was de-epithelialized without making further incisions.

**Figure 4.**
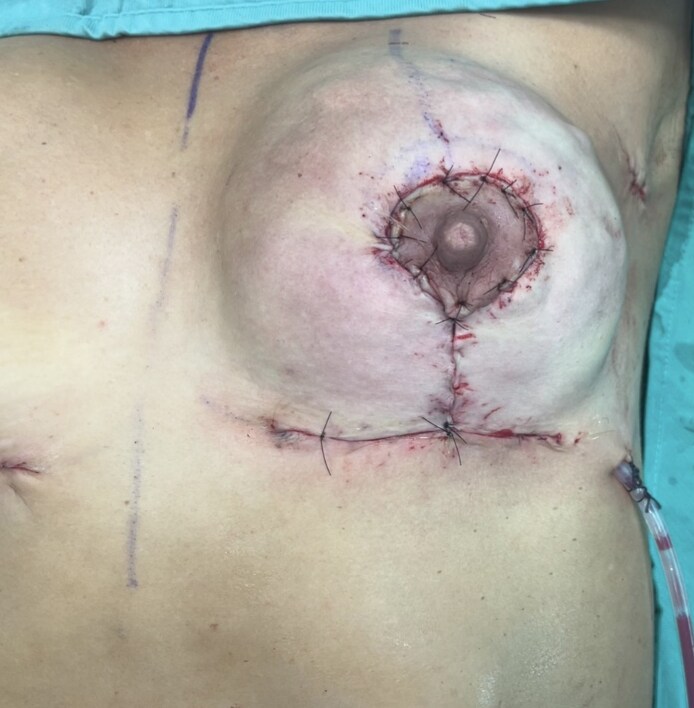
A 44-year-old female patient. The polyurethane-coated implant was inserted, and the sutures were performed.

All surgical procedures were conducted collaboratively by oncological and reconstructive surgeons under general anesthesia. Before starting the operation with the patient in a standing position, preoperative markings were made according to the rules of the Wise pattern: the NAC was repositioned by marking it along the breast meridian at the projected level of the inframammary fold on the breast's anterior surface. The 2 lateral margins of the marking were traced following the Aufricht maneuver, and after that, 2 horizontal lines were drawn at a distance from the lower side of the areola to the new inframammary sulcus between 6 and 8 cm (Figures 1, 2). The incision, where the oncology surgeon performed the mastectomy, was marked from the lower lateral triangle of the Wise pattern (Video 1). So, the oncology surgeon undertook the mastectomy from the lower lateral triangle of the Wise pattern. The dissection was performed with scissors in the superficial plane, whereas electrocautery was utilized to detach the mammary gland from the pectoral muscle. Particular care was taken to preserve tissue vascularity and avoid ischemic injury because of excessive traction. The measurements of the breast base and height were taken to identify the correct prosthesis to reconstruct a breast shape suitable for patient's anatomy (Video 2). Choosing the most suitable anatomical implant for the patient also considers symmetry in terms of shape and volume of the contralateral breast. Any necessary axillary surgery was performed through a different skin incision in the axillary. The preservation of superficial vascularization of the breast was essential to perform prepectoral reconstruction, maintaining the subcutaneous breast layer by an accurate dissection at the superficial breast fascia level. After mastectomy, a residue skin envelope of breast tissue of 1 cm was considered mandatory to perform our breast reconstruction, and the mastectomy flap had to appear well vascularized.^11^ Perfusion of mastectomy flaps was evaluated intraoperatively through clinical signs including color, temperature, bleeding from flap edges, and capillary refill without the support of indocyanine green, which was not available in our institution at the time of surgery. If the mastectomy flaps appeared too thin or not well vascularized, we proceeded intraoperatively with placement of a submuscular tissue expander. After the evaluations of thickness from the lower lateral incision of the Wise pattern, the sizer previously identified based on the measurements in the preoperative planning was positioned inside the prepectoral pocket, and it was evaluated whether the preoperative skin marking was correct or whether it was necessary to make some changes. After that, the entire portion of skin remaining within the Wise pattern design was de-epithelialized without making further incisions (Figure 3). Then the polyurethane-coated implant previously irrigated with gentamicin and vancomycin was inserted (Video 3). A suction drain was placed in the subcutaneous pocket, and it was kept in place until drainage was <20 to 25 cc per day for 2 consecutive days (Video 4). Closure was then completed at the deep dermal and subcuticular levels. We performed the intradermal suture in the horizontal and vertical portions of the inverted T and single dermal-epidermal stitches at the level of the areola (Figure 4). The perioperative antibiotic was given 30 min before the surgical incision and continued in the following days, following the recommendations of the Italian Society of Plastic Surgery (SICPRE), which advises perioperative prophylaxis for up to 72 h. A postoperative compression bra was applied immediately following surgery.

### Statistical Analysis

Demographic data (including age and BMI), as well as medical and surgical details (eg, treatments, implant size, and complications), were analyzed using descriptive statistics. Continuous data were presented as mean and range. Categorical data were summarized using counts of patients with corresponding percentages. Breast-Q scores were derived using the QScore program.

The domain-specific scores were analyzed in relation to the patients' demographic, medical, and surgical characteristics through mean values and standard deviations. Pearson's correlation coefficient was utilized to explore potential associations between BreastQ scores and continuous variables. Analysis of variance was conducted to assess differences in Breast-Q scores across groups defined by categorical variables. All tests of hypotheses were conducted at the .05 significance level.

## RESULTS

Patients' demographics, medical history, and surgical details were gathered from our prospectively maintained databases (Table 1). Patients had a mean age of 52.0 years (range, 44-63 years) and an average BMI of 24 kg/m^2^. Mean implant volume was 328 cc (from 265 to 375 cc). Forty percent of women (*n* = 4) were smokers, 1 patient received neoadjuvant chemotherapy (10%), and 6 patients received adjuvant chemotherapy (60%). Adjuvant radiotherapy was administered in 10% of patients (*n* = 1).

**Table 1.** Patient Data

Parameters	Values
No. of patients	10
No. of breasts	10
Average age, years (range)	52 (44-63)
Average BMI, kg/m^2^ (range)	24 (20-29)
No. of smokers	40% (4)
Indication for surgery	
No. of DCIS	20% (2)
No. of IDC	40% (4)
No. of ILC	40% (4)
Lymph node management	
No. of SLNB	80% (8)
No. of ALND	20% (2)
Adjuvant radiation therapy (*n*)	10% (1)
Neoadjuvant chemotherapy (*n*)	10% (1)
Adjuvant chemotherapy (*n*)	60% (6)
Average nipple-to-sternum notch distance, cm (range)	29 (26-32)
Mean hospital stay, days (range)	2 (2-2)
Average implant size, cc (range)	328 (265-375)

ALND, axillary lymph node dissection; DCIS, ductal carcinoma in situ; IDC, invasive ductal carcinoma; ILC, invasive lobular carcinoma; SLNB, sentinel lymph node biopsy.

Postoperative complications were recording during clinical evaluations at 7, 15, 30, and 90 days postsurgery and after 6 months (Table 2). Operative complications were ranked according to the Clavien–Dindo classification.^12^ Complications included mastectomy skin flap necrosis, necrosis of the NAC, surgical site infection, seroma, wound dehiscence, Grades III and IV of capsular contracture according to the Baker scale, hematoma, rippling, implant exposure, as well as displacement issues. After a 6-month follow-up, we did not observe significant complications (maximum follow-up was 18 months, the average patient follow-up time was 10 months). No cases of Grades III and IV capsular contracture occurred. We only observed 1 case of partial NAC necrosis (10%), which healed with conservative treatment, and 1 case (10%) of seromas and hematoma in the same patient, managed with drainage. We observed rippling in 3 patients (30%) after 6 months of follow-up.

**Table 2.** Surgical Complications

Complications	Values
Wound dehiscence	0
NAC necrosis	
Partial	10% (1)
Complete	0
Mastectomy skin flap necrosis	0
Prosthetic exposure	0
Surgical site infection	0
Seroma	10% (1)
Hematoma	10% (1)
Rippling	30% (3)
Capsular contracture	0
Displacement issues	0

**Figure 5.**
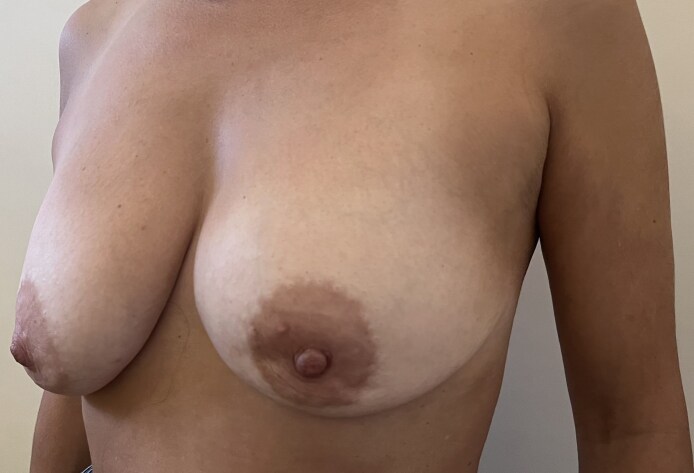
A 44-year-old female patient. Preoperative picture. Breast cancer was on the left side.

**Figure 6.**
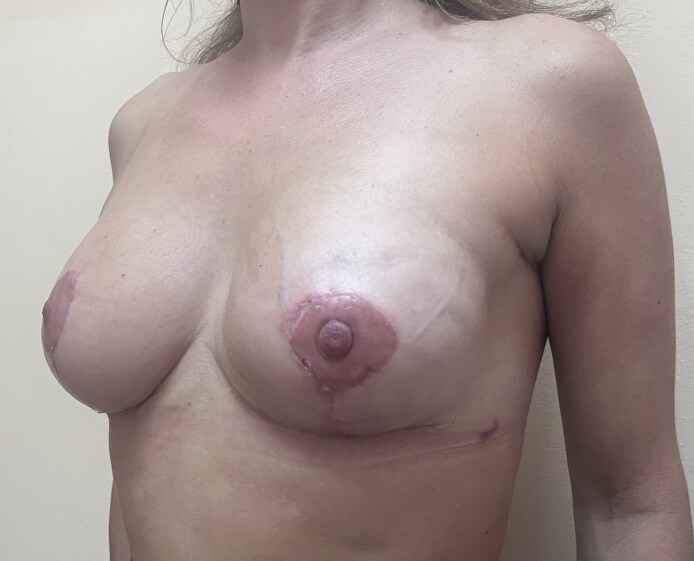
A 3-month follow-up for the 44-year-old female patient shown in Figure 5: the patient reported high satisfaction with the final results in all Breast-Q domains. We performed a nipple-sparing skin-reducing mastectomy of the left side through the lower lateral triangle of a Wise pattern with an immediate prepectoral reconstruction polyurethane-coated anatomical implant and mastopexy of the right side.

**Figure 7.**
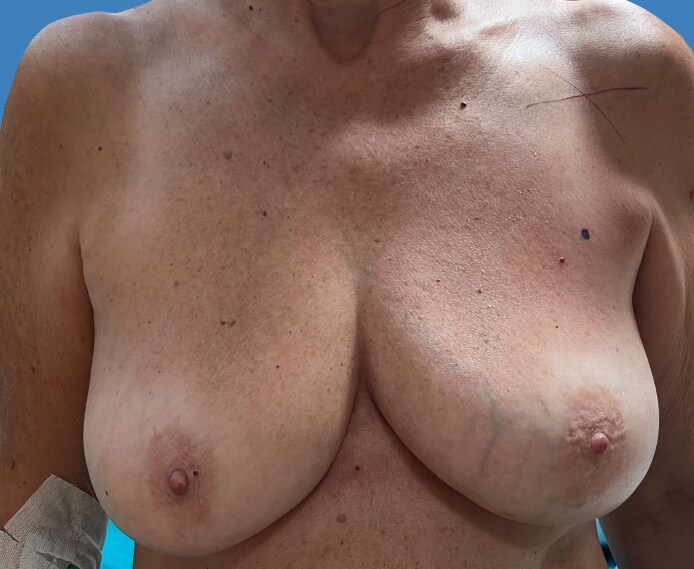
A 51-year-old female patient, preoperative picture. Breast cancer was in the left side.

**Figure 8.**
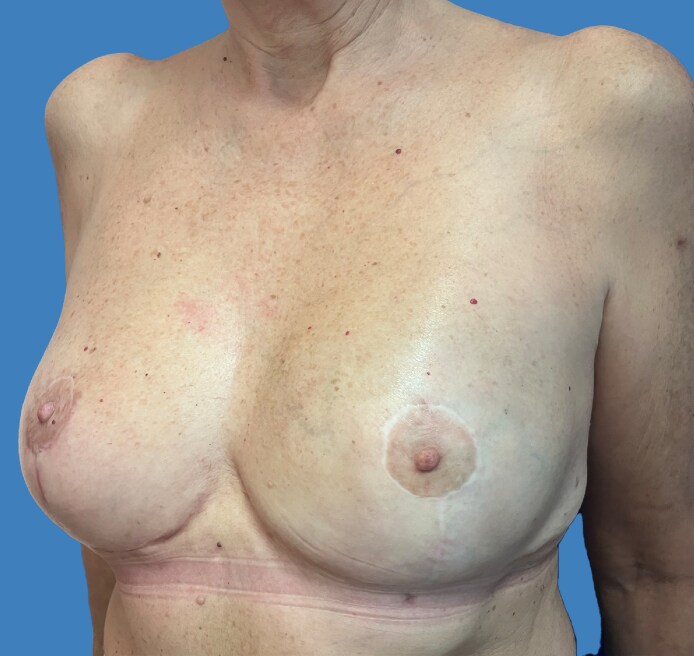
One-year follow-up for the 51-year-old female patient shown in Figure 7: the patient reported high satisfaction with the final results in all Breast-Q domains. We performed a nipple-sparing skin-reducing mastectomy of the left side through the lower lateral triangle of a Wise pattern with an immediate prepectoral reconstruction polyurethane-coated anatomical implant and breast reduction of the right side.

Breast-Q with 9 domains was reported: physical, psychosocial, and sexual well-being; breast satisfaction; outcome satisfaction; satisfaction about information provided; satisfaction with the surgeon; and satisfaction with the medical and administrative staff. Breast-Q scores vary from 0 to 100. Higher scores reflect greater satisfaction or improved health-related quality of life (Table 3). A statistical evaluation was conducted using patient characteristics and the related data on Breast-Q. Results obtained are illustrated in Tables 4 and 5. The results of the well-being domains (physical, psychological, and sexual) and the quality of life represent the average of these 3 parameters (Table 4). Greater physical well-being at Breast-Q was statistically significantly correlated with a higher BMI as well as a higher volume of the implant. Statistical analysis was also performed between the characteristics of the patients and the 3 domains of satisfaction (with the breast, outcome, and care provided), where care satisfaction was obtained as the average of satisfaction with the doctor, with staff and with information (Table 5). Greater satisfaction with the result obtained was significantly correlated with a higher BMI recorded at surgery. However, all patients reported satisfaction with the final results in all Breast-Q domains (Figures 5-8).

**Table 3.** Breast-Q

Domains	Score (range, 0-100)
Average physical well-being	82 (70-100)
Average psychosocial well-being	83 (70-90)
Average sexual well-being	75.5 (70-85)
Average satisfaction with breast	90 (80-100)
Average satisfaction with outcome	91 (80-100)
Average satisfaction with information	96 (90-100)
Average satisfaction with surgeon	100 (100-100)
Average satisfaction with the medical team	98 (90-100)
Average satisfaction with admin staff	99 (90-100)

**Table 4.** Association of Breast-Q Well-being Domains With Patient's Data (Correlation and Analysis of Variance Results)

	Physical	Psychological	Sexual	QoL
Smoke	82 (10.33)	83 (6.75)	77.5 (5.4)	80.84 (5.4)
No (*n* = 6)	81.67 (9.83)	83.33 (8.16)	76.67 (5.16)	80.56 (6.47)
Yes (*n* = 4)	82.5 (12.58)	82.5 (5)	78.75 (6.29)	81.25 (4.17)
*P*-value	.909	.8611	.5811	.8551
Cancer	82 (10.33)	83 (6.75)	77.5 (5.4)	80.84 (5.4)
DCIS (*n* = 2)	75 (7.07)	75 (7.07)	77.5 (10.61)	75.84 (8.25)
IDC (*n* = 4)	77.5 (9.57)	85 (5.77)	80 (0)	80.84 (5)
ILC (*n* = 4)	90 (8.16)	85 (5.77)	75 (5.77)	83.34 (3.85)
*P*-value	.121	.177	.4773	.3081
Lymph node	82 (10.33)	83 (6.75)	77.5 (5.4)	80.84 (5.4)
SLNB (*n* = 8)	80 (9.26)	82.5 (7.07)	76.88 (5.94)	79.79 (5.52)
ALND (*n* = 2)	90 (14.14)	85 (7.07)	80 (0)	85 (2.36)
*P*-value	.2415	.6666	.497	.2438
Radiotherapy	82 (10.33)	83 (6.75)	77.5 (5.4)	80.84 (5.4)
No (*n* = 9)	82.22 (10.93)	82.22 (6.67)	77.22 (5.65)	80.56 (5.65)
Yes (*n* = 1)	80 (NA)	90 (NA)	80 (NA)	83.33 (NA)
*P*-value	.8518	.3005	.6535	.6542
Neochemotherapy	82 (10.33)	83 (6.75)	77.5 (5.4)	80.84 (5.4)
No (*n* = 9)	82.22 (10.93)	82.22 (6.67)	77.22 (5.65)	80.56 (5.65)
Yes (*n* = 1)	80 (NA)	90 (NA)	80 (NA)	83.33 (NA)
*P*-value	.8518	.3005	.6535	.6542
Chemotherapy	82 (10.33)	83 (6.75)	77.5 (5.4)	80.84 (5.4)
No (*n* = 4)	85 (10)	85 (5.77)	77.5 (5)	82.5 (5)
Yes (*n* = 6)	80 (10.95)	81.67 (7.53)	77.5 (6.12)	79.72 (5.81)
*P*-value	.486	.477	1	.4581
Seroma	82 (10.33)	83 (6.75)	77.5 (5.4)	80.84 (5.4)
No (*n* = 9)	82.22 (10.93)	82.22 (6.67)	77.22 (5.65)	80.56 (5.65)
Yes (*n* = 1)	80 (NA)	90 (NA)	80 (NA)	83.33 (NA)
*P*-value	.8518	.3005	.6535	.6542
Hematoma	82 (10.33)	83 (6.75)	77.5 (5.4)	80.84 (5.4)
No (*n* = 9)	82.22 (10.93)	82.22 (6.67)	77.22 (5.65)	80.56 (5.65)
Yes (*n* = 1)	80 (NA)	90 (NA)	80 (NA)	83.33 (NA)
*P*-value	.8518	.3005	.6535	.6542
Rippling	82 (10.33)	83 (6.75)	77.5 (5.4)	80.84 (5.4)
No (*n* = 7)	84.29 (11.34)	82.86 (7.56)	75.71 (5.35)	80.95 (6.3)
Yes (*n* = 3)	76.67 (5.77)	83.33 (5.77)	81.67 (2.89)	80.56 (3.47)
*P*-value	.3122	.9255	.1131	.9223
Age				
Correlation	0.0781	0.2065	0.1359	0.1812
*P*-value	.8302	.5671	.7081	.6163
BMI				
Correlation	0.6664	0.3399	0.2832	0.6608
*P*-value	.0354	.3366	.4278	.0375
Nipple distance				
Correlation	−0.0589	−0.6312	0.0563	−0.2816
*P*-value	.8715	.0503	.8771	.4306
Volume implant				
Correlation	0.6478	0.4978	0.5502	0.8037
*P*–value	.0428	.1431	.0994	.0051

Significant *P*-values are highlighted in red.

ALND, axillary lymph node dissection; DCIS, ductal carcinoma in situ; IDC, invasive ductal carcinoma; ILC, invasive lobular carcinoma; NA, not applicable; QoL, quality of life; SLNB, sentinel lymph node biopsy.

**Table 5.** Association of Breast-Q Satisfaction Domains With Patient's Data (Correlation and Analysis of Variance Results)

	Satisfaction with breast	Satisfaction with outcome	Satisfaction with care
Smoke	90 (8.16)	91 (7.38)	98.25 (2.65)
No (*n* = 6)	90 (8.94)	90 (6.32)	98.33 (3.03)
Yes (*n* = 4)	90 (8.16)	92.5 (9.57)	98.13 (2.39)
*P*-value	1	.6288	.9113
Cancer	90 (8.16)	91 (7.38)	98.25 (2.65)
DCIS (*n* = 2)	90 (0)	95 (7.07)	98.75 (1.77)
IDC (*n* = 4)	82.5 (5)	85 (5.77)	99.38 (1.25)
ILC (*n* = 4)	97.5 (5)	95 (5.77)	96.88 (3.75)
*P*-value	.0078	.0949	.4423
Lymph node	90 (8.16)	91 (7.38)	98.25 (2.65)
SLNB (*n* = 8)	90 (7.56)	91.25 (6.41)	97.81 (2.81)
ALND (*n* = 2)	90 (14.14)	90 (14.14)	100 (0)
*P* value	1	.8445	.324
Radiotherapy	90 (8.16)	91 (7.38)	98.25 (2.65)
No (*n* = 9)	91.11 (7.82)	92.22 (6.67)	98.06 (2.73)
Yes (*n* = 1)	80 (NA)	80 (NA)	100 (NA)
*P*-value	.2145	.1202	.5186
Neochemotherapy	90 (8.16)	91 (7.38)	98.25 (2.65)
No (*n* = 9)	91.11 (7.82)	92.22 (6.67)	98.06 (2.73)
Yes (*n* = 1)	80 (NA)	80 (NA)	100 (NA)
*P*-value	.2145	.1202	.5186
Chemotherapy	90 (8.16)	91 (7.38)	98.25 (2.65)
No (*n* = 4)	92.5 (9.57)	90 (8.16)	98.13 (3.75)
Yes (*n* = 6)	88.33 (7.53)	91.67 (7.53)	98.33 (2.04)
*P*-value	.4619	.7483	.9113
Seroma	90 (8.16)	91 (7.38)	98.25 (2.65)
No (*n* = 9)	91.11 (7.82)	92.22 (6.67)	98.06 (2.73)
Yes (*n* = 1)	80 (NA)	80 (NA)	100 (NA)
*P*-value	.2145	.1202	.5186
Hematoma	90 (8.16)	91 (7.38)	98.25 (2.65)
No (*n* = 9)	91.11 (7.82)	92.22 (6.67)	98.06 (2.73)
Yes (*n* = 1)	80 (NA)	80 (NA)	100 (NA)
*P*-value	.2145	.1202	.5186
Rippling	90 (8.16)	91 (7.38)	98.25 (2.65)
No (*n* = 7)	92.86 (7.56)	92.86 (4.88)	97.86 (3.04)
Yes (*n* = 3)	83.33 (5.77)	86.67 (11.55)	99.17 (1.44)
*P*-value	.0899	.2453	.5063
Age			
Correlation	0.2187	0.5324	−0.0592
*P*-value	.5439	.1131	.871
BMI			
Correlation	0.6556	0.6737	−0.2527
*P*-value	.0396	.0327	.4813
Nipple distance			
Correlation	0.2236	0.5774	0.1149
*P*-value	.5346	.0805	.752
Volume implant			
Correlation	0.2696	0.1849	0.3033
*P*-value	.4514	.609	.3942

Significant *P*-values are highlighted in red.

ALND, axillary lymph node dissection; DCIS, ductal carcinoma in situ; IDC, invasive ductal carcinoma; ILC, invasive lobular carcinoma; NA, not applicable; SLNB, sentinel lymph node biopsy.

## DISCUSSION

Mastectomy continues to be frequently required for the treatment of breast malignancies. An immediate reconstruction through a single-step breast implant offers the possibility to restore breast shape and volume harmonious with the body with a noninvasive and technically simple procedure.^13^ In the case of medium-large and ptotic breasts, the removal of excess skin after mastectomy becomes necessary to perform a prepectoral direct-to-implant. Such reduction allows reshaping of the breast envelope, also obtaining an improvement of the breast appearance compared with the presurgery condition. Also, the body image is better with a lift of the NAC in the correct place in case of a ptotic breast after breast reconstruction. However, it is essential to preserve vascularization for the NAC as much as possible.

In prepectoral breast reconstruction, interface materials are considered a prerequisite for state of the art, including preventing capsular contracture. ADM is frequently utilized but is expensive and associated with complications. Alternatively, a silicone implant coated with polyurethane foam can be utilized. In a variety of studies, polyurethane coating tended to be associated with fewer short-term complications than ADM, including seroma and infection, and the silicone implant coated with polyurethane is associated with a low incidence of capsular contracture, absence of implant rotation, and late seroma.^14-16^ One-stage immediate breast reconstruction with an anatomical implant with polyurethane covers after mastectomy appears to be oncologically safe, also showing a high level of patient satisfaction.^17^ In order to quantify the level of patient satisfaction after breast surgery, Breast-Q was developed for breast reconstruction.^18^

The strengths of our technique are listed below:

- It is a single-stage breast reconstruction using anatomical implants, without the need to do a 2-stage reconstruction and without the use of ADM for reconstruction.
- It is a prepectoral reconstruction without the need to lift the pectoral muscle, which allows quicker recovery in terms of pain and convalescence.
- It is possible to correct breast ptosis by having a breast with a shape and size that is more suited to the chest and characteristics of the patient.
- Preservation of the NAC is achieved without the need for a graft, therefore resulting in an improved cosmetic outcome.
- The risk of necrosis of the mastectomy flaps and of the NAC is drastically reduced, even in active smokers, because access from the lower lateral triangle of the Wise pattern allows maintaining almost all the vascular connections (medial, superior, inferior, and lateral), in particular the fifth and the second intercostal perforators.^19^
- The surgical access to perform the mastectomy is adequate and safe to remove the entire gland, even in the presence of medium-large and ptotic breasts.
- It is a quick procedure and not expensive procedure.
- It is a technique that satisfies both the patient and the surgeon regarding the aesthetic aspect, having minimal impact on the psychological aspect of the patient. Greater physical well-being at Breast-Q was statistically significantly correlated with a higher BMI as well as a higher volume of the implant.
- It is possible to symmetrize the other breast at the same time as the mastectomy with mastopexy or breast reduction.

Unfortunately, our study has some limitations: the number of cases is small, and the Breast-Q questionnaire was not performed before the surgery. Lipofilling, a quick and straightforward procedure, will be necessary a few months later to resolve rippling. However, there is no animation deformity pain or functional limitation, given that the reconstruction is in the prepectoral plane.^20-22^

In the literature, there are many surgical techniques for breast reconstruction, both autologous and heterologous, but the trend is toward single-stage prepectoral reconstruction because of its benefits both in terms of complications and patient satisfaction.^23-27^ Some authors have reported their experience of breast reconstruction regarding skin reshaping after mastectomy in large and ptotic breasts.^28,29^

Breast surgery involves a multidisciplinary approach, with many specialists collaborating to guarantee the patient the best care and reconstruction.

## CONCLUSIONS

Our experience with mastectomy from the lower lateral triangle of the Wise pattern, combined with direct prepectoral reconstruction using polyurethane-covered anatomical implants, suggests that this novel approach may offer a valuable option for women with medium-to-large and ptotic breasts. Our technique appears to allow immediate prepectoral reconstruction while potentially minimizing the risks of skin necrosis associated with skin reduction and maintaining an appropriate NAC position. Early results indicate that it could be a reproducible method leading to aesthetically pleasing outcomes and satisfaction for both patients and surgeons. Nevertheless, larger studies with longer follow-up are needed to confirm these findings and to compare this approach with other reconstructive techniques in breast ptosis.

## References

[1] Kontos M, Lanitis S, Constantinidou A, et al Nipple-sparing skin-reducing mastectomy with reconstruction for large ptotic breasts. J Plast Reconstr Aesthet Surg. 2020;73:690–695. doi: 10.1016/j.bjps.2019.11.025 31928958

[2] Nava MB, Cortinovis U, Ottolenghi J, et al Skin-reducing mastectomy. Plast Reconstr Surg. 2006;118:603–610. doi: 10.1097/01.prs.0000233024.08392.14 16932166

[3] Newman MK. Reconstruction of the ptotic breast using Wise pattern skin deepithelialization. Plast Reconstr Surg Glob Open. 2016;4:e1077. doi: 10.1097/GOX.0000000000001077 27975010 PMC5142476

[4] Caputo GG, Pisano G, Albanese R, et al Immediate prepectoral implant-based breast reconstruction after J-pattern skin-reducing mastectomy. Plast Reconstr Surg. 2024;154:233e–236e. doi: 10.1097/PRS.0000000000011028 37647526

[5] De Vita R, Buccheri EM, Villanucci A, Pozzi M. Breast reconstruction actualized in nipple-sparing mastectomy and direct-to-implant, prepectoral polyurethane positioning: early experience and preliminary results. Clin Breast Cancer. 2019;19:e358–e3363. doi: 10.1016/j.clbc.2018.12.015 30691930

[6] Gschwantler-Kaulich D, Leser C, Salama M, Singer CF. Direct-to-implant breast reconstruction: higher complication rate vs cosmetic benefits. Breast J. 2018;24:957–964. doi: 10.1111/tbj.13113 30230119

[7] Liu J, Zheng X, Lin S, Han H, Xu C. A systematic review and meta-analysis on the prepectoral single-stage breast reconstruction. Support Care Cancer. 2022;30:5659–5668. doi: 10.1007/s00520-022-06919-5 35182228

[8] Seth AK, Sisco M. Prepectoral breast reconstruction. Plast Reconstr Surg. 2025;155:213e–227e. doi: 10.1097/PRS.0000000000011737 39700251

[9] Pusic AL, Klassen AF, Scott AM, Klok JA, Cordeiro PG, Cano SJ. Development of a new patient-reported outcome measure for breast surgery: the BREAST-Q. Plast Reconstr Surg. 2009;124:345–353. doi: 10.1097/PRS.0b013e3181aee807 19644246

[10] Regnault P. Breast ptosis. Definition and treatment. Clin Plast Surg. 1976;3:193–203. doi: 10.1016/S0094-1298(20)30220-0 1261176

[11] Rancati A, Angrigiani C, Hammond D, et al Preoperative digital mammography imaging in conservative mastectomy and immediate reconstruction. Gland Surg. 2016;5:9–14. doi: 10.3978/j.issn.2227-684X.2015.08.01 26855903 PMC4716857

[12] Dindo D, Demartines N, Clavien PA. Classification of surgical complications: a new proposal with evaluation in a cohort of 6336 patients and results of a survey. Ann Surg. 2004;240:205–213. doi: 10.1097/01.sla.0000133083.54934.ae 15273542 PMC1360123

[13] Antoniazzi E, De Grazia A, Dell'Antonia F, et al Immediate prepectoral breast reconstruction in nipple-sparing mastectomy with Wise-pattern incision in large and ptotic breasts: our experience and short-term results. J Plast Reconstr Aesthet Surg. 2024;91:154–163. doi: 10.1016/j.bjps.2024.01.042 38412604

[14] Correia-Pinto JM, Poleri F, Barbosa JP, et al Comparing polyurethane and acellular dermal matrix implant cover in prepectoral breast reconstruction: short-term complications. Plast Reconstr Surg Glob Open. 2023;11:e4798. doi: 10.1097/GOX.0000000000004798 36751508 PMC9894346

[15] Loreti A, Siri G, De Carli M, et al Immediate breast reconstruction after mastectomy with polyurethane implants versus textured implants: a retrospective study with focus on capsular contracture. Breast. 2020;54:127–132. doi: 10.1016/j.breast.2020.09.009 33010626 PMC7529839

[16] Vázquez G. Patients' satisfaction with anatomic polyurethane implants. Gland Surg. 2017;6:185–192. doi: 10.21037/gs.2016.11.02 28497022 PMC5409901

[17] Rancati A, Soderini A, Dorr J, Gercovich G, Tessari L, Gonzalez E. One-step breast reconstruction with polyurethane-covered implants after skin-sparing mastectomy. J Plast Reconstr Aesthet Surg. 2013;66:1671–1675. doi: 10.1016/j.bjps.2013.07.005 23932524

[18] Pusic AL, Chen CM, Cano S, et al Measuring quality of life in cosmetic and reconstructive breast surgery: a systematic review of patient-reported outcomes instruments. Plast Reconstr Surg. 2007;120:823–837. doi: 10.1097/01.prs.0000278162.82906.81 17805107

[19] Nahabedian MY, Angrigiani C, Rancati A, Irigo M, Acquaviva J, Rancati A. The importance of fifth anterior intercostal vessels following nipple-sparing mastectomy. Plast Reconstr Surg. 2022;149:559–566. doi: 10.1097/PRS.0000000000008828 35006210

[20] Caputo GG, Mura S, Contessi Negrini F, Albanese R, Parodi PC. From sub-pectoral to Pre-pectoral implant reconstruction: a decisional algorithm to optimise outcomes of breast replacement surgery. Healthcare (Basel). 2023;11:671. doi: 10.3390/healthcare11050671 36900676 PMC10001132

[21] Ribuffo D, Berna G, De Vita R, et al Dual-plane retro-pectoral versus Pre-pectoral DTI breast reconstruction: an Italian multicenter experience. Aesthetic Plast Surg. 2021;45:51–60. doi: 10.1007/s00266-020-01892-y 32860077 PMC7886728

[22] Cogliandro A, Salzillo R, De Bernardis R, et al Prepectoral versus subpectoral direct-to-implant breast reconstruction: evaluation of patient's quality of life and satisfaction with BREAST-Q. Aesthetic Plast Surg. 2023;47:1291–1299. doi: 10.1007/s00266-023-03316-z 36944866

[23] King CA, Bartholomew AJ, Sosin M, et al A critical appraisal of late complications of prepectoral versus subpectoral breast reconstruction following nipple-sparing mastectomy. Ann Surg Oncol. 2021;28:9150–9158. doi: 10.1245/s10434-021-10085-z 34386913

[24] Domenici L, Caputo GG, Losco L, et al Muscle-sparing skin-reducing breast reconstruction with pre-pectoral implants in breast cancer patients: long-term assessment of patients' satisfaction and quality of life. J Invest Surg. 2022;35:841–847. doi: 10.1080/08941939.2021.1923874 34015977

[25] Susini P, Nisi G, Pierazzi DM, et al Advances on capsular contracture-prevention and management strategies: a narrative review of the literature. Plast Reconstr Surg Glob Open. 2023;11:e5034. doi: 10.1097/GOX.0000000000005034 37305202 PMC10256414

[26] Li Y, Xu G, Yu N, Huang J, Long X. Prepectoral versus subpectoral implant-based breast reconstruction: a meta-analysis. Ann Plast Surg. 2020;85:437–447. doi: 10.1097/SAP.0000000000002190 31913902

[27] Salgarello M, Visconti G, Barone-Adesi L. Current trends in breast reconstruction. Minerva Surg. 2021;76:526–537. doi: 10.23736/S2724-5691.21.08987-5 34935321

[28] Cordova A, Rossi M, Roggio T, et al The wide base bipedicled (WIBB) flap in nipple-sparing skin-reducing mastectomy. Sci Rep. 2024;14:9226. doi: 10.1038/s41598-024-52396-7 38649704 PMC11035620

[29] La Padula S, Pensato R, Al-Amer R, et al Three pedicle-based nipple-sparing skin-reducing mastectomy combined with prepectoral implant-based breast reconstruction. Plast Reconstr Surg. 2024;154:430e–441e. doi: 10.1097/PRS.0000000000011092 37749785

